# First Report of *Bartonella* spp. in Marsupials from Brazil, with a Description of *Bartonella harrusi* sp. nov. and a New Proposal for the Taxonomic Reclassification of Species of the Genus *Bartonella*

**DOI:** 10.3390/microorganisms10081609

**Published:** 2022-08-09

**Authors:** Renan Bressianini do Amaral, Marita Vedovelli Cardozo, Alessandro de Mello Varani, Maria Eduarda Chiaradia Furquim, Clara Morato Dias, William Oliveira de Assis, Alanderson Rodrigues da Silva, Heitor Miraglia Herrera, Rosangela Zacarias Machado, Marcos Rogério André

**Affiliations:** 1 Programa de Pós-Graduação em Microbiologia Agropecuária, Faculdade de Ciências Agrárias e Veterinárias, Universidade Estadual Paulista (UNESP), Jaboticabal 15385-000, SP, Brazil; 2 Laboratório de Imunoparasitologia, Departamento de Patologia, Reprodução e Saúde Única, Faculdade de Ciências Agrárias e Veterinárias, Universidade Estadual Paulista (UNESP), Jaboticabal 14884-900, SP, Brazil; 3 Departamento de Biotecnologia Agropecuária e Ambiental, Faculdade de Ciências Agrárias e Veterinárias, Universidade Estadual Paulista (UNESP), Jaboticabal 14884-900, SP, Brazil; 4 Laboratório de Biologia Parasitária, Universidade Católica Dom Bosco, Campo Grande 79117-900, MS, Brazil

**Keywords:** bartonelosis, marsupialia, biochemical characterization, phylogenomics, WGS, taxonomic classification

## Abstract

The genus *Bartonella* (Rhizobiales: Bartonellaceae) encompasses facultative intracellular Gram-negative alphaproteobacteria that parasitize mainly erythrocytes and endothelial cells, as well as macrophages, monocytes and dendritic cells. Although they can infect numerous mammal species and arthropod vectors worldwide, reports of *Bartonella* infections in marsupials are scarce. In fact, such agents have only been detected in marsupials and/or associated ectoparasites in Australia and the United States of America until the present moment. The present study aimed to isolate and characterize molecularly, morphologically and phenotypically *Bartonella* infecting free-living marsupials sampled in the Brazilian Pantanal, the largest wetland in South America. Two marsupials were captured in December 2018 and six marsupials in February 2019, totaling eight small mammals sampled: five (62.5%) *Thylamys macrurus* and three (37.5%) *Monodelphis domestica*. All blood samples were submitted to qPCR for *Bartonella* spp. based on the *nuoG* gene, a pre-enrichment liquid culture and a chocolate agar solid culture. *Bartonella* sp. was isolated from 3 *T. macrurus* and one *M. domestica*. One *Bartonella* isolate obtained from a *T. macrurus* blood sample (strain 117A) that showed to be closely related to the *Bartonella vinsonii* complex and *Bartonella machadoae* was selected for whole genome sequencing using a hybrid approach based on Illumina NovaSeq and Nanopore sequencing platforms. This strain showed a genome of 2.35 Mbp, with an average C + G content of 38.8%, coding for 2013 genes, and a 29 kb plasmid with an average C + G content of 34.5%. In addition, this strain exhibited an average nucleotide identity (ANI) of 85% with *Bartonella* species belonging to the *B. vinsonii* group and 91% with *B. machadoae*. Phylogenomic analysis based on 291 protein coding genes shared by the genomes of 53 *Bartonella* species positioned this strain closely to *B. machadoae*. This new isolated species was named *Bartonella harrusi* sp. nov., which was characterized as having small capnophilic, microaerophilic and aerobic rods with an absence of pili and flagella. In conclusion, the present work describes the biochemical, phenotypic and genomic characteristics of *Bartonella harrusi*, a new species isolated from the *T. macrurus* blood samples of the Brazilian Pantanal. Finally, a review of the taxonomic classification of members of the genus *Bartonella* is proposed, based on the ANI values accessed by whole genome sequencing analyses.

## 1. Introduction

The *Bartonella* genus comprise Gram-negative, intracellular facultative and fastidious α-2-Proteobacteria [1,2]. These bacteria are known to infect a wide range of vertebrates worldwide, mostly mammals, and may cause different clinical manifestations depending on the host and species of *Bartonella* involved [3]. These agents are transmitted by hematophagous arthropods (fleas, ticks, mosquitoes, flies and lice) [4,5].

Currently, this genus comprises 37 species, among which some are represented by more than one subspecies, validated according to the “List of Prokaryotic names with Standing in Nomenclature database” (https://lpsn.dsmz.de/genus/bartonella—accessed on 1 June 2022) [6,7,8,9,10]. The vast majority of the validated *Bartonella* species were submitted to biochemical, phenotypic and molecular characterization by DNA–DNA hybridization techniques or whole genome sequencing (List of Prokaryotic names with Standing in Nomenclature database). In view of the availability of collections of prokaryotic genomes, bioinformatics approaches have become robust tools to trace taxonomic and phylogenetic relationships between organisms.

Marsupials are viviparous mammals belonging to the Infraclass Marsupialia with the completion of development in the marsupial [11]. In Brazil, 58 species of marsupials have been described, most of them are small in size and with mainly arboreal habits and can be found in all Brazilian biomes (https://www.icmbio.gov.br/portal/faunabrasileira/estado-de-conservacao/2802-mamiferos-marsupiais—accessed on 1 June 2022). Although *Bartonella* spp. is widely studied in felids, rodents and bats, there are few studies on the detection, molecular characterization and/or isolation of *Bartonella* in marsupials. In fact, to date, only one species of *Bartonella* (*Bartonella australis*) has been isolated from kangaroos (*Macropus giganteus*) in Australia [12]. Additionally, a *Bartonella* sp. phylogenetically related to *B. tamiae* was detected in *Ixodes tasmani* ticks collected from koalas (*Phascolarctos cinereus*) in Australia [13]. Additionally, ‘*Candidatus Bartonella bandicootii*’ and ‘*Candidatus Bartonella woyliei*’ were detected in fleas (*Pygiopsylla tunneyi*) of “bandicoots” (*Perameles bougainville*) and fleas (*Pygiopsylla hilli*) and ticks (*Ixodes australiensis*) collected from woylies (*BIettongia penicillata*), respectively [7]. In the Americas, *Bartonella* spp., closely related to *B. henselae* and *B. clarridgeiae*, were molecularly detected in *Ctenocephalides felis* fleas collected from opossums (*Didelphis virginiana*) in the USA [14,15]. In Brazil, scarce are the studies on the occurrence of *Bartonella* spp. in marsupials. In fact, only three studies were found in the literature regarding the molecular detection of *Bartonella* spp. in this group of mammals. Based on conventional and real-time PCR assays, *Bartonella* DNA was not found in marsupials’ blood samples in the states of Mato Grosso do Sul and Rio de Janeiro [16,17,18].

The present study aimed to detect and characterize, using microbiological, molecular and bioinformatics analyses, *Bartonella* sp. in blood samples from wild marsupials sampled in the Pantanal biome, a flooded area in central-western Brazil. Additionally, a new isolate of *Bartonella* from *Thylamys macrurus* was characterized, using morphological, biochemical, phenotypic and molecular characters. Finally, by using bioinformatics analyses of the whole genomes of the *Bartonella* species deposited in international databases, the present study aimed to contribute to the re-assessment of the separation of the *Bartonella* species using ANI values, as well as to infer differences in the metabolic profile of this group of agents.

## 2. Material and Methods

### 2.1. Ethics Statement

The procedures and management protocols involving the capture and blood sampling of wild marsupials were approved by the “Chico Mendes Institute for Biodiversity Conservation” (SISBIO number 64204) and the “Ethics Committee on Animal Use of the Faculty of Agrarian and Veterinary Sciences” (FCAV/UNESP) (protocol number #11794).

### 2.2. Area of Study and Collection of Samples

Wild marsupials were captured during two expeditions in the Pantanal of the state of Mato Grosso do Sul, central-western Brazil, more specifically in the “Alegria” farm, Nhecolândia region (−19.050399; −56.675623): one in December 2018 and the second in February 2019. The procedures performed to obtain biological samples of the small mammals were described in a previous work [10].

**Figure 1:**
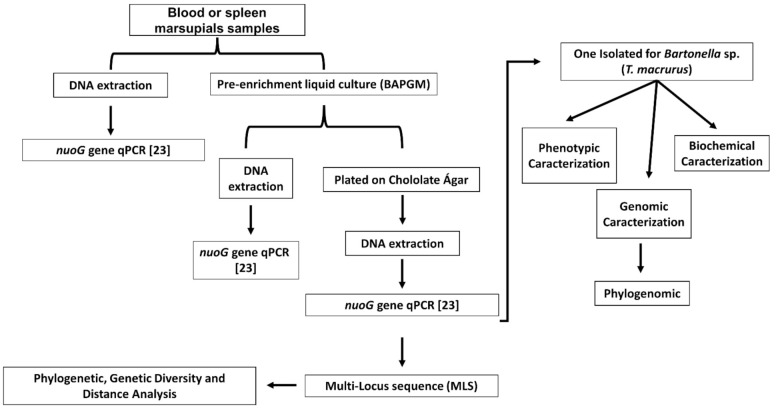
Flowchart of the microbiological and molecular analyses used in this study for the isolation and characterization of *Bartonella harrusi* sp. nov.

The blood samples collected from the marsupials were stored in DNAse/RNAse-free microtubes containing ethylenediaminetetraacetic acid (EDTA) and were kept in liquid nitrogen cylinders. Once in the laboratory they were stored in a freezer −80 °C (Figure 1).

### 2.3. Bartonella Isolation

A pre-enrichment liquid medium previously described as *Bartonella*-Alphaproteobacteria Growth Medium (BAPGM) was used in the present study. Briefly, 500 mL of IPL-41 insect medium (Sigma-Aldrich, St. Louis, MO, USA) were supplemented as previously described [10,19,20,21]. Two vials of negative controls were also added: one containing only the pre-enrichment medium and the other one containing both the pre-enrichment medium and sheep defibrinated blood [10,21].

After seven days, 200 μL of liquid culture were submitted to DNA extraction with the Illustra Tissue and Cells genomic Prep Mini Spin kit, following the manufacturer’s recommendations. Additionally, 200 μL of the contents of each bottle were seeded onto an enriched chocolate agar (Laborclin, Pinhais, Paraná, Brazil) and kept at 35 °C with 5% CO_2_ for up to 60 days in a CO_2_/O_2_ Water Jacketed incubator (NuAire, Plymouth, MA, USA) and observed daily. Three negative controls were used: two corresponding to the negative controls previously used in the liquid culture of pre-enrichment and the other represented by a chocolate agar plate without any biological samples. Smooth and circular colonies suggestive of *Bartonella* were collected and submitted to DNA extraction by the boiling method [22] and qPCR assay for *Bartonella* sp. based on the *nuoG* gene [23]. Colonies molecularly confirmed as belonging to the genus *Bartonella* were then submitted to five passages until pure isolates were obtained.

### 2.4. Quantitative Real-Time PCR Assay (qPCR) for Bartonella sp. Based on the nuoG Gene (Nicotinamide Adenine Dinucleotide Dehydrogenase Gamma Subunit) and Conventional PCR (PCR) for the Mammalian Endogenous Gene

DNA from the marsupial blood samples was extracted using the InstaGene™ Matrix kit (Bio-Rad Laboratories, São Paulo, Brazil), according to the manufacturer’s recommendations. A PCR assay based on the mammalian endogenous glyceraldehyde gene 3-phosphate dehydrogenase (*gapdh*) [24] was used to verify the absence of inhibitors in the DNA samples extracted. A quantitative PCR protocol (qPCR) based on the *nuoG* gene [23] was used to detect and quantify *Bartonella* sp. DNA (the number of copies of a 83 bp fragment of *nuoG* gene/μL) in the marsupial blood samples, marsupial blood samples added to the BAPGM-based liquid culture and colonies isolated from the chocolate agar plates. PCR assays were conducted on low profile multiplate PCR plates (BioRad™, Hercules, CA, USA) using CFX96 thermocycler (BioRad™, Hercules, CA, USA). The standard curves were constructed with serial dilutions of plasmid DNA (pIDTSMART—Integrated DNA Technologies) (1.0 × 10^7^ to 1.0 × 10^0^ copies/μL), which encoded an 83 pb fragment of the *Bartonella henselae nuoG* gene. The number of copies of the plasmid was determined by (Xg/μL DNA/[plasmid length in pb × 660]) × 6.022 × 10^23^ × plasmid/μL copies [23] All the DNA samples were initially tested in duplicates. All duplicates whose difference in Cq (the cycle of quantification) values was higher than 0.5 were retested in triplicates.

### 2.5. Multilocus Analysis for Bartonella sp.

For molecular characterization, isolates that were positive in the qPCR for *Bartonella* sp. were submitted to previously described conventional PCR assays based on six molecular markers: 16S rRNA (400 bp) [25], *gltA* (750 bp) [26], the 16S-23SrRNA ITS intergenic spacer region (453-717pb) [20], *groEL* (752pb) [27], *ftsZ* (600pb) [27] and *rpoB* (800bp) [27]. *Bartonella henselae* previously detected in a cat sampled in southeastern Brazil [21] and sterile ultrapure water (Nuclease-Free Water, Promega™, Madison, WI, USA) were used as positive and negative controls, respectively. The amplicons obtained in the conventional PCR assays were electrophoresed in agarose gels (1%) stained with ethidium bromide (Life Technologies, Carlsbad, CA, USA) at 100 V and 150 mA for 50 min. The gels were photographed under ultraviolet light (ChemiDoc MP Imaging System; Bio Rad, Hercules, CA, USA).

### 2.6. Concatenated Phylogenetic Analysis Based on the 16S rRNA, gltA, groEL, ftsZ, rpoB Genes and ITS Intergenic Region

Products amplified in conventional PCR assays were purified using ExoSAP-IT (Thermo Fisher Scientific, Waltham, MA, USA) and sequenced using the BigDye™ Terminator v3.1 Cycle Sequencing Kit (Thermo Fisher Scientific™, Waltham, MA, USA) and the ABI PRISM 310 DNA Analyzer (Applied Biosystems™, Foster City, CA, USA) [28]. The primers used in the sequencing reactions were the same used in the conventional PCR assays for *Bartonella* sp. The sequences obtained were first submitted to a screening test using the Phred–Phrap software version 23 [29,30] to assess the quality of the electropherogram and to obtain consensus sequences from the alignment of the forward and reverse sequences.

The BLAST program (BLASTn) [31] was used to compare the obtained nucleotide sequences with those from the international database (GenBank) [32]. The consensus sequences obtained in this study and those retrieved from GenBank were aligned using the Clustal/W software [33] via Bioedit v. 7.0.5.3 [34]. Phylogenetic analysis was based on the Maximum Likelihood (ML) method and was inferred with the W-IQ-Tree tool available online (http://iqtree.cibiv.univie.ac.at/—accessed on 12 December 2021) [35] using 1000 bootstrap replicates. The best evolutionary model was selected by the jModelTest2 program (version 2.1.6) in XSEDE [36]. The tree was edited in Treegraph 2.0.56–381 beta [37].

In addition, nucleotide sequences were aligned with sequences previously deposited in GenBank and were subjected to a Split-Network-based distance analysis using SplitsTree v4.11.3 software [38].

### 2.7. Phenotypic Characterization of Bartonella sp.

For phenotypic characterization, a *Bartonella* isolated from a blood sample of *Thylamys macrurus* (strain 117A) was seeded on chocolate agar plates (Novamed) and incubated at 35 °C in duplicates, under the following conditions: (i) aerobic (i.e., standard incubator); (ii) capnophilic (i.e., 5% CO_2_) in a CO_2_ incubator (Thermo Forma II Water Jacket CO_2_ Incubator, Thermo Scientific); (iii) microaerophilic (i.e., 5 to 15% O_2_, 10% CO_2_) using Microaerobac^®^ bags (Probac); and (iv) anaerobic (i.e., <3% O_2_, 4 to 10% CO_2_) using Anaerobac^®^ bags (Probac).

### 2.8. Light Microscopy and Scanning Electron Microscopy (SEM)

The *Bartonella* strain 117A was submitted to light microscopy and scanning electron microscopy (SEM). For light microscopy, the isolate was stained with Gram (Hy-labs) and visualized using 100× objectives. For SEM, the *Bartonella* isolate (strain 117A) was collected from the chocolate agar plate and fixed in a solution containing 2% paraformaldehyde, 2.5% glutaraldehyde and 0.1 M cacodylate buffer for 1 h and then washed three times in a phosphate buffer. Then, 1% osmium tetroxide was added and the solution was incubated for 30 min. The bacteria were then dehydrated through a graded ethanol series (50–100%) and dried with a critical point dryer (EMS 850). After the critical point, the sample was fixed in metal stubs, and coated with gold (5 nm) in a Danton Vacuum Desk II. Finally, the bacterial cells were visualized by SEM. The samples were analyzed and photographed using a Zeiss Evo 10 scanning electron microscope (10–20 kV).

### 2.9. Biochemical and Metabolic Characterization

For biochemical and metabolic characterization, the *Bartonella* strain 117A isolated from *T. macrurus* was tested with the identification systems BBL Crystal Enteric/Nonfermenter (BD Becton Dickinson, Franklin Lakes, NJ, USA), API 20E and NF III (Probac), following the manufacturers’ instructions. The enzymatic catalase reaction was carried out with hydrogen peroxide (3%).

### 2.10. Growth Curve

To perform the growth curve of the *Bartonella* isolated from the marsupial (strain 117A), 10 colony-forming units (CFU) were removed from the chocolate agar plates and deposited into 500 mL of a supplemented BAPGM medium. The inoculum was then maintained at 35 °C with 5% CO_2_ in a CO_2_/O_2_ culture incubator (Water Jacketed, NuAire, Plymouth, USA). Every 6 h, 100 µL were collected for 8 days, totaling 32 collections. This volume was subjected to a 9:1 serial dilution in a supplemented BAPGM medium (10^−1^ to 10^−6^). One hundred microliters of each of the six dilutions were plated onto chocolate agar plates with a Drigalski loop. Bacterial quantification was performed using the standard plate counting method, determining the number of colony-forming units (CFU/mL). Each plate was stored at 35 °C with 5% CO_2_ in a CO_2_/O_2_ culture incubator (Water Jacketed, NuAire). The incubation period of the plates was 72 h and, after this period, colony-forming units per mL (CFUs/mL) were counted.

### 2.11. Whole Genome Sequencing of Bartonella Isolated from Blood Samples of Thylamys Macrurus (Strain 117A) and Genome Annotation

The genomic DNA extraction from the *Bartonella* isolate of *T. macrurus* (strain 117A) previously grown on chocolate agar plates was performed using the commercial kit DNeasy^®^ Blood and Tissue (QIAGEN, Hilden, Germany), following the manufacturers’ instructions. For genome assembly, the genomic DNA sample was sequenced by Illumina Novaseq 6000 (PE 2 × 150 bp). A total of 5,871,200 paired-end reads were obtained. These reads were trimmed with Trimmomatic [39]. In addition, FASTQ files were also obtained from a MinION run (kit SQK-LSK109, Oxford Nanopore, Oxford, UK), totaling 118,191 reads, with an average read length of 5471 bp. The readings were clipped to a low quality and to adapters using NanoFilt and Porechop, respectively [40]. All the trimmed Illumina and Nanopore reads were then used for hybrid assembly using the MaSuRCa v4.0.4 assembler with default parameters [41]. The assembled genome was automatically annotated with the NCBI Prokaryotic Genome Annotation Pipeline [42].

### 2.12. Phylogenomic Analyses

The assembled genome of the new *Bartonella* isolate (strain 117A) and 53 complete *Bartonella* genomes retrieved from the NCBI RefSeq database [43] were used for the comparative analysis. For this purpose, 291 shared genes were retrieved using the roary tool [44]. These genes were aligned with MAFFT [45] and were later used for phylogenomic analyses.

An ML analysis was inferred with the W-IQ-Tree tool [35] using 1000 bootstrapping replicates. The trees were edited in Treegraph 2.0.56-381 beta [37]. *Brucella abortus* and *Brucella ceti* (GenBank accession number: CP007681; NC_022905) were used as an outgroup.

The average nucleotide identities (ANI) between *Bartonella harrusi* sp. Nov., *Bartonella machadoae* and the species of the *Bartonella vinsonii* complex (*B. vinsonii* subsp. *Vinsonii*, *B. vinsonii* subsp. *Arupensis* and *B. vinsonii* subsp. *Berkhoffii*) were calculated with OrthoANIu 1.2 [46]. In addition, an ANI analysis was performed between the nucleotide sequence of the plasmid found in *Bartonella harrusi* compared with eight other plasmid sequences from the *Bartonella* species deposited in GenBank.

### 2.13. Taxonomic Reclassification of Bartonella Species Based on ANI

The whole genome consensus sequence of the new *Bartonella* isolate (strain 117A) and the 216 whole genomes of the *Bartonella* species retrieved from the NCBI RefSeq database [43] were submitted to the dRep pipeline [47]. After filtering by length, the completeness and contamination of the sequences were analyzed using CheckM [48]. Primary and secondary clustering was performed using pairwise MASH, in order to calculate the ANI. As ANI cut-off parameters, 95% and 97% were used for primary and secondary clustering, respectively, aiming at separating the species and assigning objective taxonomic classifications to be applied to all the available genomes of the *Bartonella* species.

### 2.14. “In Silico” Analysis of the Metabolic Profile and Ribosomal and Polymerase Phylogenetic Markers for Bartonella Species

The assembled whole genome of the new *Bartonella* isolate (strain 117A) and 53 whole genomes of the *Bartonella* species retrieved from the NCBI RefSeq database [43] were aligned by the RASTtk (Rapid Annotations using Subsystems Technology) pipeline [49]. After annotation, a GenomeSet was generated, which was used to analyze the metabolic profile and search for phylogenetic markers via the HMMER tool available online (https://www.kbase.us/—accessed on 12 December 2021) [50].

### 2.15. Search for Active Carbohydrate Enzymes (CAZy) Families

A search for the occurrence of carbohydrate active enzymes (CAZy) families was performed. For this purpose, the GenomeSet described in the section above was submitted to the dbCAN database for the automated annotation of the active carbohydrate enzymes [51] using HMMER 3 via the HMMER tool available online (https://www.kbase.us/—accessed on 12 December 2021) [50].

## 3. Results

### 3.1. Occurrence of Bartonella sp. in Marsupial Blood Samples

Two marsupials were captured in December 2018 and six marsupials in February 2019. Among the eight marsupials sampled, five (62.5%) animals belonged to the species *Thylamys macrurus* and three (37.5%) to the species *Monodephis domestica*. All eight marsupial blood DNA samples were positive in the PCR for the endogenous mammalian *gapdh* gene. None of the eight DNA samples extracted from marsupial blood were positive in the qPCR for *Bartonella* sp. based on the *nuoG* gene. The efficiency, R^2^, slope and intercept on the Y axis of the reactions ranged from 91.4% to 103.9%, 0.995 to 0.999, −3.547 to −3252 and 38.571 to 40, respectively (Table 1).

### 3.2. Isolation of Bartonella sp. in Chocolate Agar

Out of the eight marsupial blood samples submitted to the pre-enrichment liquid BAPGM culture, four (50%; two *T. macrurus* blood samples and two *M. domestica* blood samples) were positive in the qPCR for *Bartonella* sp. The number of copies of the *nuoG* gene fragment per microliter ranged from 5.9 × 10^2^ to 2.93 × 10^6^. The efficiency, R^2^, slope and intercept on the Y axis of the reactions ranged from 91.4% to 103.9%, 0.995 to 0.999, −3.547 to −3252 and 38.571 to 40, respectively.

Out of the four qPCR-positive liquid culture samples, three (45.16%) (two *T. macrurus* blood samples and one *M. domestica* blood sample) showed the presence of *Bartonella* colonies on chocolate agar plates, whose identity was confirmed by qPCR for *Bartonella* spp. based on the *nuoG* gene (Table 1).

### 3.3. Multilocus and Phylogenetic Analyses for Bartonella sp.

A BLASTn analysis demonstrated that the different target genes of the *Bartonella* colonies isolated from marsupials showed high identities to *B. machadoae* and *Bartonella* genotypes previously reported in ectoparasites collected from rodents from Brazil (Table 2).

**Figure 2:**
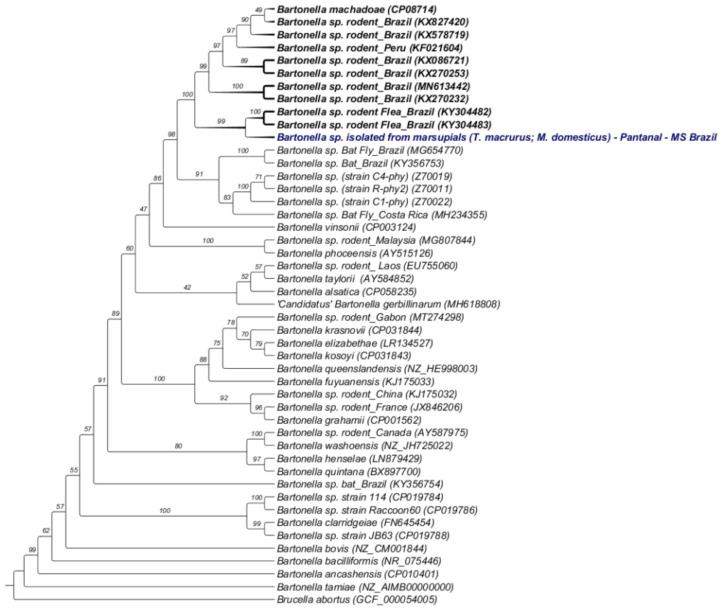
Concatenated phylogenetic analysis within the genus *Bartonella* based on the 16S rRNA, *gltA*, *ftsZ*, *groEL*, *rpoB* genes and 16S-23S rRNA intergenic region (ITS). The phylogenetic tree was inferred using the Maximum Likelihood method and the evolutionary model TVM + F + I. The sequences obtained in this study are highlighted in red. The numbers in each branch correspond to the bootstrap values accessed with 1000 replicates. *Brucella abortus* was used as an outgroup.

The phylogenetic tree based on the sequences of the 16S rRNA*, gltA, groEL, ftsZ, rpoB* genes and ITS intergenic region of three *Bartonella* isolates from one *T. macrurus* individual (strains 117A, 117B and 117C) and one from *M. domestica* (strain 78A) inferred by the Maximum Likelihood method (ML) and evolutionary model TVM + F + I formed a single and monophyletic cluster. The sequences of *Bartonella* isolated from marsupials were positioned together with sequences previously detected in *Polygenis* (*P*.) *bohlsi bohlsi* fleas collected from rodents in the Pantanal, state of Mato Grosso do Sul (KY304482, KY304483) and in a larger clade with several sequences of *Bartonella* sp. previously detected in *Oecomys mamorae* rodents in the Brazilian Pantanal, state of Mato Grosso do Sul (KX578719, KX827420), rodents from the Brazilian states of Maranhão, Ceará and Rio de Janeiro (KX086721, KX270253, MN613442, KX270232, MN613441, KX270248), rodent-associated genotypes from Peru (KF021604) and *B. machadoae* (CP08714). Furthermore, this clade was closely related to the clade of *Bartonella* genotypes detected in bats and Streblidae bat flies (*Strebla guajiro*) (MG654770, KY356753) and *Bartonella* sp. previously detected in Cricetidae rodents from Peru and the USA (C4-phy, R-phy2, C1-phy) (Figure 2).

### 3.4. Diversity Analyses

**Figure 3:**
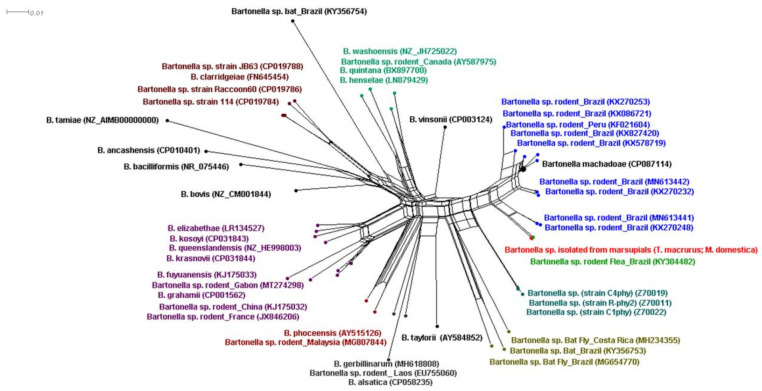
Neighbor-Net analysis of DNA sequences from the 16S rRNA, *gltA, ftsZ, groEL, rpoB* genes and 16S-23S intergenic region (ITS) of *Bartonella* sp. isolated from wild marsupials (*T. macrurus* strain 117A, 117B and 117C and *M. domestica* strain 78A) sampled in the Brazilian Pantanal in the present study when compared to closely related *Bartonella* sequences previously deposited in GenBank.

The nucleotide sequences (16S rRNA, *gltA*, *groEL*, *ftsZ*, *rpoB* and ITS) obtained from four *Bartonella* isolates from two marsupials, one from the *T. macrurus* species (strains 117A, 117B and 117C) and one from the *M. domestica* species (strain 78A), were identical to each other. The genealogical analysis of the nucleotide sequences by Network, using the program Splitstree v4.11.3 [38], showed a similar result to that obtained by the phylogenetic analysis, forming three relevant groups. Cluster #1, consisting of sequences from *Bartonella* isolates of *T. macrurus* and *M. domestica*, was related to *Bartonella* genotypes previously detected in rodent-associated fleas in the Pantanal (MKY304482; KF021604). Cluster #2 consisted of *Bartonella* genotypes previously detected in rodents from the Brazilian states of Mato Grosso do Sul, Maranhão, Ceará and Rio de Janeiro (KX578719, KX578720, KX086721, KX270253, MN613442, KX270232, MN613441, KX270248), rodents from Peru (KF021604) and *B. machadoae*. Cluster #3 contained *Bartonella* genotypes previously detected in rodents from Peru and the USA (Figure 3).

### 3.5. Phenotypic and Morphological Characterization of Bartonella sp.

**Figure 4:**
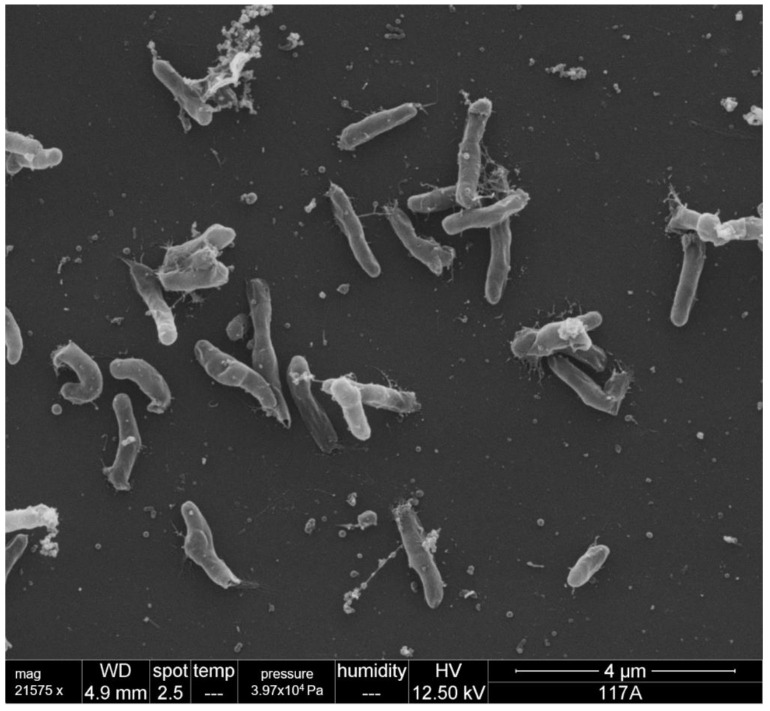
Scanning electron microscopy (SEM) of *Bartonella* strain 117A isolated from a blood sample of *T. macrurus* (*Bartonella harrusi* sp. nov.) showing absence of flagellum and pili. A 4 µM scale bar is shown in the image.

The *Bartonella* 117A strain isolated from a blood sample of *T. macrurus* was characterized by Gram-negative rods 1–2.0 µm long and 0.3–0.6 µm wide, without flagella and pili. The bacteria proved to be capnophilic, microaerophilic and aerobic, since there was no growth under anaerobic conditions. In SEM, the bacteria appeared as small rods without polar pili and flagella, forming compact aggregates of bacteria (Figure 4 and Figure S1).

### 3.6. Biochemical and Metabolic Characterization

The *Bartonella* strain 117A obtained from the blood sample of *T. macrurus* was negative for oxidase, catalase, urease, indole production, nitrate reduction and acetoin production (Voges–Proskauer reaction). It was inert for all the tested carbohydrates (glucose, mannitol, inositol, sorbitol, rhamnose, sucrose, melibiose, amygdalin and arabinose) and negative for arginine, lysine and tryptophan arylamidase activities. Furthermore, it was negative for esculin, DNAse activity, β-D-galactosidase nitrate and McConkey growth. It showed sensitivity to Polymyxin B. 3.6.

### 3.7. Growth Curve

**Figure 5:**
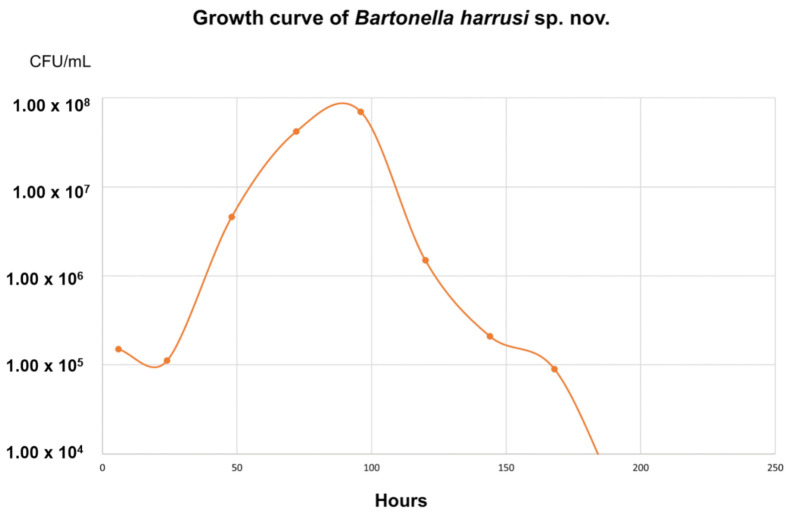
Graph of the growth curve of *Bartonella harrusi* sp. nov. representing the collection hours (*X* axis) by the quantification of CFU/mL (*Y* axis).

The *B. harrusi* growth curve showed moderately slow growth in the supplemented BAPGM medium. The bacteria entered the logarithmic phase 30 h after the inoculum preparation and maintained an average quantification of 10^7^ CFU/mL until 90 h of the experiment. After this period, the bacteria entered the decline phase (Figure 5).

### 3.8. Phylogenomic Analysis

**Figure 6:**
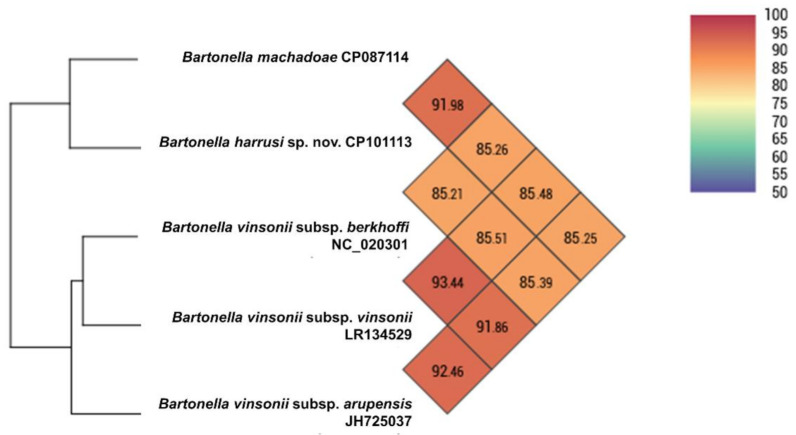
Heat map generated with OrthoANI values calculated between A: *Bartonella harrusi* sp. nov. isolated from a blood sample of *T. macrurus*, *B. machadoae*, species of the *B. vinsonii* complex.

**Figure 7:**
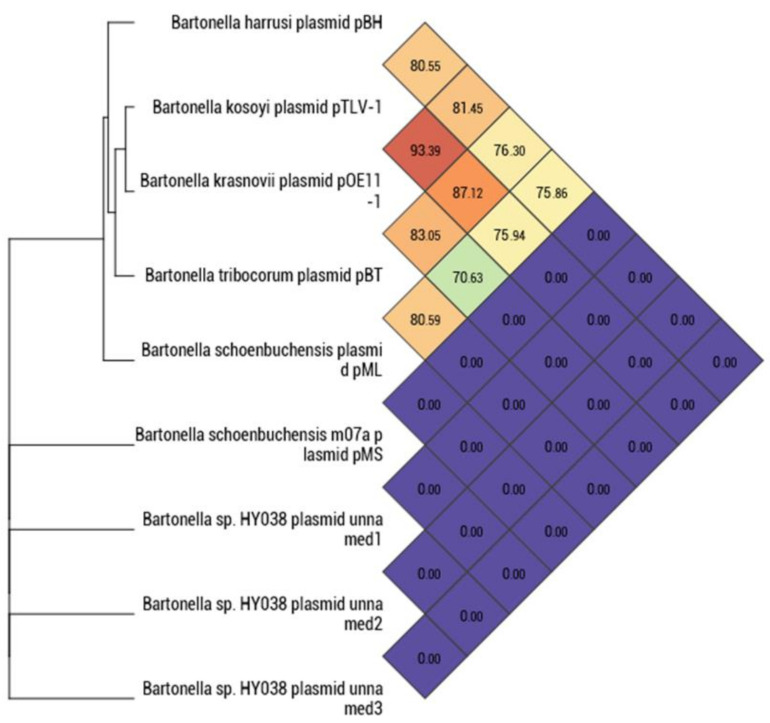
Heat map generated with OrthoANI values calculated between the plasmids found in *Bartonella* species and the plasmid found in *Bartonella harussi* sp. nov. isolated in the present study using the OAT software Tool version 0.93.1 [46].

The genome was assembled into a 2.23 Mbp circular chromosome and a 29 Kbp circular plasmid. The genomic characteristics of the *Bartonella* strain 117A are described in Table 3. The whole genome ANI of strain 117A was calculated in relation to phylogenetically related species: ANI values of 85% and 91.98% were found when compared to *Bartonella* species belonging to the *B. vinsonii* complex (*B. vinsonii* subsp. *vinsonii*, *B. vinsonii* subsp. *arupensis* and *B. vinsonii* subsp. *berkhoffii*) and *B. machadoae*, respectively (Figure 6). In the ANI analysis of the plasmid sequences of the *Bartonella* species available in GenBank, the *Bartonella* strain 117A showed 81.45% when compared to *Bartonella krasnovii*, which was the highest value found. Plasmids of the *Bartonella* sp. strain HY038 and the pMS plasmid from *B. schoenbuchensis* did not show any phylogenetic relationship with the plasmids found in other *Bartonella* species (Figure 7).

**Figure 8:**
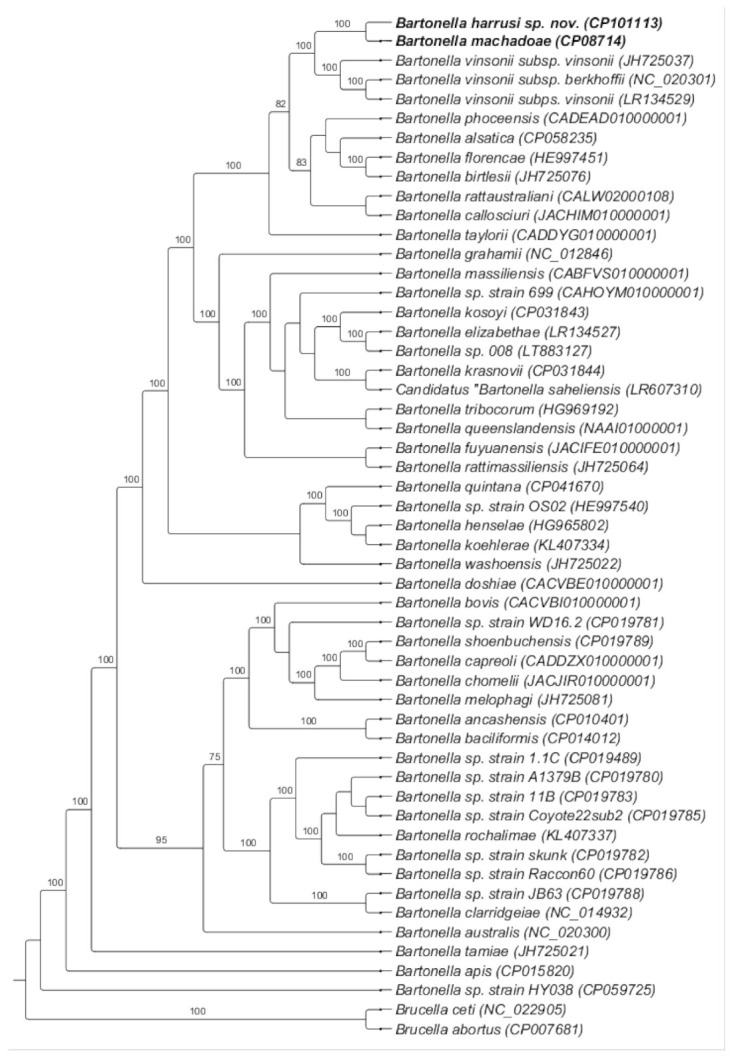
Phylogeny based on the complete genome of 53 species of *Bartonella*, using 293 coding genes shared between these species. Bootstrap values are shown under nodes. *Brucella abortus* and *Brucella ceti* were used as an outgroup.

The phylogenomic analysis was based on 291 protein-coding genes shared by 53 whole genomes of *Bartonella* species with validly published names and some *Bartonella* species not yet named. Based on this analysis, the *Bartonella* strain 117A was positioned in a clade closely related to *B. machadoae* and *Bartonella* species belonging to the *B. vinsonii* complex, albeit separated with 100% bootstrap (Figure 8).

### 3.9. Use of Average Nucleotide Identity (ANI) to Separate Species of the Genus Bartonella

**Figure 9:**
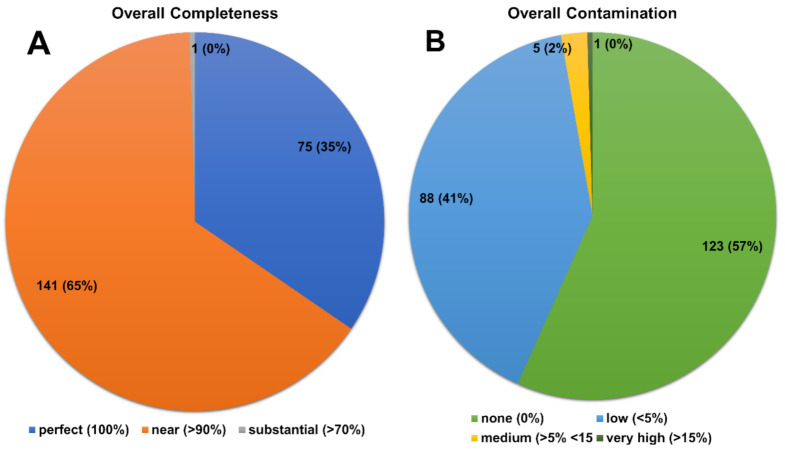
Pie chart showing (**A**) the completeness of the 217 analyzed genomes of *Bartonella* spp. and (**B**) the level of contamination of the genomes used for the analysis of ANI.

The *Bartonella* genomic sequences available in Genbank were analyzed for completeness and contamination in order to determine the quality of the genomes for use in ANI-based taxonomic classification. Among the 217 genomes available, only the genome of ‘*Candidatus Bartonella saheliensis*’ (LR607310) presented a low quality both for completeness (below 70%) and percentages of contamination (above 15%) (Figure 9A,B), and it was therefore removed from subsequent analyses. Regarding the mean nucleotide identity (ANI) in general, most *Bartonella* species showed ANI values between 75 and 90%. However, ANI values lower than 70% were found for the *B. tamiae*, *B. apis* and *Bartonella* sp. strain HY038, species considered more diversified within this genus when compared to the other *Bartonella* species analyzed (Figure S2). Furthermore, it was possible to observe ANI values greater than 95% among the available *Bartonella* genomes, which were divided into 50 different groups (Figure S2 and Table S1). Among these groups, 20 were represented by a single sequence and 30 had more than one sequence with a variation between 95 and 100%. Genomes with more than one sequence were subdivided using cut-off points 95 < ANI < 97%. Eighteen groups presented ANI values above 97% when compared to the other genomes; 12 genomes had one to four sequences with variations between 95 to 97% of ANI, and they were reclassified as subspecies (Table S1).

### 3.10. “In Silico” Analysis of Metabolic Profiles, Ribosomal and Polymerase Phylogenetic Markers for Species of the Genus Bartonella

The analysis of the metabolic profile for species of the genus *Bartonella* using the genome available in Genbank showed similar profiles: all species analyzed (n = 53) had the capacity to oxidize hydrogen and oxygen, as well as to fix carbon. Some *Bartonella* species (strain 117A, *B. machadoae*, *B. tribocorum*, *B. schoenbuchensis*, *B. massiliensis*, *B. capreoli*, *B. ancashensis*, *B. bovis*, *B. bacilliformis*, *B. rattimassiliensis* and *B. melophagi*) were not able to oxidize sulfur because they did not have related genes. The most diverse species found were the *Bartonella* sp. Strain HY038 and *Bartonella apis*, which presented in their genomes genes related to nitrogen fixation, the oxidation of urea, selenium, arsenic and halogenated compounds, a profile that was not observed in any other *Bartonella* species. Interestingly, the *Bartonella* sp. strain HY038, *Bartonella apis* and *Bartonella tamiae* showed genes related to the use of C1 compounds (Figure S3).

Regarding the profile of ribosomal phylogenomic markers and polymerases, only punctual differences were found. In fact, none of the analyzed species showed considerable differences (Figure S4).

### 3.11. Search for Active Carbohydrate Enzymes (CAZy) Families

When analyzing genes related to groups of enzymes, Carbohydrate Esterases (CAZy), enzymes classified into families 24, 94 and 103 of the group of glycoside hydrolases enzymes, families 19, 29, 30, 51 and 84 of the group of glycoside transferases, and family 11 of the group of carbohydrate transferases were found in all the analyzed genomes (Figure S5). However, genes related to the production of family 3 of enzymes of the glycoside hydrolases group were found only in some *Bartonella* species belonging to lineages 1 (*B. tamiae*, *B. apis*, *B. ancachensis* and the *Bartonella* sp. strain HY038), 2 (*B. bovis*, *B. schoenbuchensis* and *B. chomelii*) and 3 (*B. clarridgeiae*, *B. rochalimae* and the *Bartonella* spp. strains Coyote22, stunk WD16.2 and 1-1C JB63). Family 4 of the group of glycoside transferases was found only in a few species, including *B. machadoae* recently isolated from rodents in the same region where the present study was performed, but not in the *Bartonella* sp. strain 117A described in the present study (Figure S5).

Interestingly, although family 24 of glycoside hydrolases was found in the genome of the 53 *Bartonella* species analyzed, there was a difference in the number of occurrences depending on the analyzed genome, with variations from 1 to 12 genic regions (Figure S5).

## 4. Discussion

The present work aimed to contribute to the diagnosis, molecular, morphological and phenotypic characterization of *Bartonella* spp. in wild marsupials from the Pantanal, state of Mato Grosso do Sul, central-western Brazil.

There are few works in the literature that have investigated the role of marsupials as reservoirs of *Bartonella* spp. For instance, Fournier et al. [12] isolated and described *Bartonella australis* in a kangaroo (*Macropus giganteus*) in Australia. *Bartonella* DNA was also detected in *Ixodes tasmani* ticks collected from koalas (*Phascolarctos cinereus*) [13] in Australia. Additionally, ‘*Candidatus Bartonella bandicootii*’ and ‘*Candidatus Bartonella woyliei*’ were molecularly detected in fleas (*Pygiopsylla tunneyi*) from bandicoots (*Perameles bougainville*) and in fleas (*Pygiopsylla hilli*) and ticks (*Ixodes australiensis*) collected from woylies (*BIettongia penicillata*), respectively [7]. When it comes to Brazil, *Bartonella* DNA was not detected in marsupials (*Thylamys macrurus*, *Gracilinanus agilis*, *Monodelphis domestica* and *Didelphis albiventris*) in the Pantanal of Mato Grosso do Sul [16]. This finding might be associated with the low *Bartonella* bacteremia, which might have hampered the detection of this agent by qPCR directly from the blood. The *Bartonella* bacteremia in the marsupials sampled by [16] was probably below the limit of detection of the qPCR technique based on the *nuoG* gene. Similar results were found herein when marsupial blood DNA samples were submitted to qPCR assays. In the present study, *Bartonella* DNA was found in marsupial blood samples only after passage in a BAPGM liquid medium (a pre-enriched liquid culture) and the isolation of *Bartonella* spp. on a chocolate agar. The present work brings the first detection and isolation of *Bartonella* in marsupials in the Americas.

The use of a BAPGM pre-enrichment medium and isolation on a chocolate agar brings greater sensitivity in the diagnosis of *Bartonella* spp., as previously reported in [19] where, when using the abovementioned diagnostic platform for *Bartonella* spp. in dog blood samples, they found a positivity of 8.1%; on the other hand, when they performed a molecular diagnosis directly from the blood DNA samples, a positivity of 4.7% was found. Our research group has been using this diagnostic platform and showing the advantages of using the “liquid medium” for the successful diagnosis and isolation of *Bartonella henselae* in blood samples from cats [21]. More recently, using the same diagnostic platform used in the present study, we reported the diagnosis and isolation of *Bartonella machadoae* from rodent blood samples sampled in the Pantanal of Mato Grosso do Sul [10].

Regarding the phenotypic characteristics, the strain 117A of *Bartonella* sp. isolated from a blood sample of *T. macrurus* showed microbiological and morphological characteristics identical to the *B. machadoae* recently described by our research group [10], showing excellent growth under conditions of increased CO_2_. Additionally, this strain showed aerobic, capnophilic and microaerophilic characteristics. It was characterized by rounded and smooth colonies (1–2 mm) after 4–5 days of plating on a chocolate agar. Similarly to *B. machadoae*, the strain 117A was not able to grow under anaerobic conditions. Such growth characteristics are similar to those reported in other species of the genus *Bartonella*, such as in *B. elizabethae*, *B. henselae* (the Houston-3 strain), *B. clarridgeiae*, *B. quintana* and *B. vinsonii* subsp. *Vinsonii* [52]. In contrast, *B. kosoyi* and *B. krasnovii* isolated from *Rattus rattus* and *Synosternus cleopatrae* fleas collected from *Gerbillus andersoni*, respectively, did not grow under strictly aerobic and anaerobic conditions [53].

The *Bartonella* strain 117A was negative for oxidase, catalase, urease, indole production, acetoin production and nitrate reduction. These characteristics were similar to those found in the biochemical profiles of the *Bartonella* species of the *B. vinsonii* complex [54] and *B. machadoae* [10]. However, differences were found with other *Bartonella* species: while *B. krasnovii* was able to ferment sucrose, citrate and arabinose, *Bartonella kosoyi* was able to ferment sorbitol, melibiose, rhamnose and glucose, in addition to showing a positive reaction to arginine [2]. On the other hand, but similarly to *B. machadoae*, *Bartonella harrusi* sp. nov. was not able to ferment the abovementioned carbohydrates [10].

Regarding the bacterial growth curve, the results of the present study showed discrepancies between the growth curves of the two isolated species: while *B. machadoae* (unpublished data) required a shorter time to enter into the logarithmic phase and presented a higher peak of CFU/mL, remaining longer in the stationary phase, the *Bartonella* strain 117A needed more time to reach the highest CFU/mL count, in addition to remaining in the stationary phase for a shorter time. When compared with other species of the genus, *B. henselae* (using a supplemented RPMI 1640 medium) and *B. quintana* (using a DS2 medium formulated to favor the cell growth of insect cells) showed curves similar to that presented by the *Bartonella* strain 117A, both requiring a longer time to enter into the logarithmic phase and, similarly to the present work, they did not remain in a stationary phase for a long time [55].

The Bartonellae isolated in the present study did not show pili or flagella at SEM, which was confirmed by the absence of genes encoding adhesin (*BadA*) and flagella (*FlaA* and *FlaB*). The absence of pili or flagellum is shared with other rodent-associated *Bartonella* species, such as *B. vinsonii* [56], *B. japonica*, *B. silvatica* [57], *B. heixianziensis* [58] and *B. machadoae* [10]. Differently, *B. bacilliformis*, *B. schoenbuchensis*, *B. capreoli*, *B. chomelii*, *B. clarridgeiae* and *B. rochalimae* have flagella. The presence of pili has been described in rodent-associated *Bartonella* spp. such as *B. tribocorum* [59], *B. alsatica* [60], *B. acomydis*, *B. jaculi*, *B callosciuri*, *B. pachyuromydis* [61], *B. fuyuanensis* [58], *B. kosoyi* and *B. krasnovii* [2].

With regard to multilocus analysis (MLSA), do Amaral et al. [10] demonstrated that, due to the high genotypic diversity of the *Bartonella* species described in different hosts, previously used criteria, such as those proposed by la Scola et al. [62], have less accuracy and precision for the discrimination of strictly related *Bartonella* species. In the present work, it was possible to observe a lack of resolution of the phylogeny based on six molecular markers, which were able to separate the *Bartonella* sp. isolated from marsupials in the present work from the *Bartonella* species belonging to the *B. vinsonii* complex, but were not able to clearly separate them from *B. machadoae*, a rodent-associated *Bartonella* species recently described in the same region where the present study was performed [10] (Figure 2; Table 4).

Phenotypic and biochemical analyses are shown to be inefficient in the discrimination of new species of *Bartonella* due to very similar biochemical profiles, with rare exceptions in more diversified species, as shown in the present study by “in silico” analyses. A Multilocus analysis based on 6–8 molecular markers was replaced by a WGS analysis, due to greater ease and more accessible values of WGS of the *Bartonella* isolates. In fact, phylogenomic analyses based on hundreds of genes provide higher reliability in the definition of species, in addition to allowing the inference of biochemical profiles without the use of time-consuming and laborious techniques [10,63].

In the present study, a bioinformatics analysis applied to whole genomes of the *Bartonella* species deposited in databases allowed us to find a relative diversity in genes related to CAZy families, such as the presence of family 3 in some *Bartonella* species and the variations in the amount of occurrences of glycosyl hydrolases in family 24, enzymes known as β-N-acetylglucosaminidase and muramidase, respectively, and related to the degradation of chitin-based carbohydrates [64]. More studies related to CAZy families should be conducted, since chitinases may play a role in the interaction of bartonellae and the peritrophic membrane of ectoparasites, enabling bartonella mobility and favoring transmission. Furthermore, it was possible to identify genes related to family 4 glycosyltransferase enzymes in some species of *Bartonella*. Considering that such enzymes are associated with the accumulation of glycogen for the survival of the agent in the environment, they may be involved in the maintenance of some *Bartonella* species in flea and lice feces [65]. Interestingly, although no genes related to this family of enzymes were found in the genome of the *Bartonella* strain 117A isolated herein, such genes were found in the genome of *B. machadoae*, which was recently isolated from *Trichomys fosteri*’ blood samples in the same region where the present study was performed [10]. Thus, the role of this enzyme family in the survival and transmission of *Bartonella* species between their vertebrate and invertebrate hosts should be further investigated.

Although the number of species and Candidatus for the genus *Bartonella* has increased in the last years, especially those associated with small mammals, many of them have not been fully characterized and, quite often, with insufficient information for the reliable determination of new species and subspecies [63]. In addition to demonstrating the inability to distinguish phylogenetically close species by the criteria previously used, the present work proposes a review of the taxonomic classification of species of the genus *Bartonella* by using ANI values obtained from whole genomes. ANI values below 95% are used as a cut-off point for determining new species [2,66,67]. Based on this criterion, it is possible to separate the genus *Bartonella* into 50 different species (Table S1). In addition, we were able to solve taxonomic issues in several subgroups of the genus *Bartonella*. For instance, the taxa *Bartonella apis* and *Bartonella tribocorum* are composed of two and three different species, respectively. *Bartonella* subspecies belonging to the *Bartonella vinsonii* complex actually comprise three different species (*Bartonella vinsonii*, *Bartonella arupensis* and *Bartonella berkhofhii*), since they have an ANI value below 95% among themselves. On the other hand, *B. schoenbuchensis*, *Bartonella chomelii*, *B. capreoli* and *B. melophagi* should be grouped as a subspecies of *B. schoenbuchensis* (the species described first among the four cited), since they show ANI values above 95% among themselves. Finally, based on the *Bartonella* species (*B. henselae*, *B. quintana* and *B. bacilliformis*) with the highest number of genomes deposited in the database, the variation of the ANI values within the same species ranged from 97 to 100%. Therefore, we propose a cut-off point of 95 < ANI < 97% for the classification of new *Bartonella* subspecies (Table S1).

The present work brings the first description of *Bartonella* in marsupials outside the Australian continent. We propose that it be named *Bartonella harrusi* sp. nov., in honor of Israeli researcher Shimon Harrus, professor at The Hebrew University of Jerusalem, Koret School of Veterinary Medicine, for his extensive contribution to the study of members of the *Bartonella* genus.

Despite the vectors involved in the transmission of this new *Bartonella* species are still unkown, siphonapterans may act as possible vectors of this agent. Indeed, *gltA* sequences from *B. harrusi* sp. nov. showed 100% identity with *Bartonella* genotypes previously detected in rodent-associated fleas (*Polygenis* (*Polygenis*) *bohlsi bolshi*, *Polygenis occidentalis occidentalis*, *Polygenis platensis* and *Craneopsylla minerva minerva)* in the biomes of Pantanal (the state of Mato Grosso do Sul), Pampa and the Atlantic Forest, in the state of Rio Grande do Sul [16,68]. Interestingly, the *Bartonella* genotypes detected in fleas from central-western and southern Brazil were found in regions where the circulation of *B. machadoae*, a species recently described in rodents, was also reported [10]. Recently, Santana et al. [69] detected *B. machadoae* in *Amblyomma sculptum* ticks collected from wild boars (*Sus scrofa*) in southeastern Brazil. Thus, it is necessary to evaluate the diversity of hosts, vectors and the zoonotic potential of *B. machadoae* and *B. harrusi*, considering that both are closely related to *Bartonella* species belonging to the *B. vinsonii* complex, which have already been shown to have zoonotic potential in several regions in the world [6,10,54,70,71,72].

### Description of Bartonella harrusi sp. nov.

The bacterial cells of this new proposed species are rods (1–2.0 µm long and 0.3–0.6 µm wide) without flagella and pili, showing excellent growth under capnophilic conditions (5% CO_2_) at 35 °C. Colonies grown on chocolate agar are smooth and circular. The time required for growth to reach colonies of 1.0–2.0 mm is 4 days at 35 °C. They are negative for oxidase, catalase, urease, indole production, acetoin production and nitrate reduction. They are unable to ferment the carbohydrates sorbitol, melibiose, rhamnose and glucose, albeit they showed a positive reaction to arginine. The type strain (117A) carried a single circular chromosome of 2.23 MB, with an average G + C DNA content of 38.8% by mol. and a 29,892 bp circular plasmid with an average G + C content of 34.5%. The closest species with a valid published name is *B. machadoae* (91.98% ANI). *Bartonella harrusi* sp. nov. (strain 117A) is stored at the Laboratory of Immunoparasitology, Department of Pathology, Reproduction and Single Health, Faculty of Agrarian and Veterinary Sciences/Universidade Estadual Paulista, UNESP, Jaboticabal, SP, Brazil.

## 5. Conclusions

The present work presents the first description of *Bartonella* in marsupials from the Americas. *Bartonella harrusi* sp. nov. was isolated from blood samples of *T. macrurus* in the Pantanal of Mato Grosso do Sul. The diagnostic platform combining qPCR results based on the *nuoG* gene from blood samples in a pre-enrichment liquid culture and chocolate agar isolation proved to be efficient in detecting *Bartonella* in marsupials. A phylogenomic analysis based on 291 genes and an ANI value of 91.98% obtained by the WGS of *Bartonella* 117A allowed the separation of *Bartonella harrusi* sp. nov. from *Bartonella machadoae*. A bioinformatics analysis applied to complete genomes should be used for the classification and taxonomic review of members of the genus *Bartonella* based on ANI values.

## Figures and Tables

**Table 1:** Blood samples from marsupials captured in the Brazilian Pantanal and subjected to a qPCR for *Bartonella* sp. based on the *nuoG* gene (with quantification of the target gene fragment/µL), liquid culture in BAPGM pre-enrichment medium and isolation on chocolate agar plates.

		qPCR for *Bartonella* spp. (Number of *nuoG* Copies/µL)	
Marsupial Species (ID)	Blood	Liquid Culture (BAPGM)	Solid Culture (Chocolate Agar)
*Thylamys macrurus* **(#22)**	Neg	Neg	Neg
*Thylamys macrurus* **(#36)**	Neg	Neg	Neg
*Monodelphis domestica* **(#70)**	Neg	Neg	Neg
*Monodelphis domestica* **(#78)**	Neg	1.39 × 10^5^	**Pos**
*Thylamys macrurus* **(#79)**	Neg	Neg	Neg
*Monodelphis domestica* **(#96)**	Neg	2.16 × 10^7^	**Pos**
*Thylamys macrurus* **(#116)**	Neg	1.37 × 10^5^	**Pos**
*Thylamys macrurus* **(#117)**	Neg	5.9 × 10^2^	**Pos**

(#) sample identification.

**Table 2:** BLASTn results for six target gene sequences from four *Bartonella* isolates obtained from marsupial blood samples (three from *T. macrurus* and one from *M. domestica*).

Target Gene	Species	Host	Locality	Identity	Query Cover	“E-Value”	GenBank Accession Number
16S rRNA	*Bartonella* sp. strain R-phy1	Não descrito	France	99.16%	100%	1 × 10^−179^	Z70005
*gltA*	*Bartonella* sp.	*Polygenis* (*P*.) *bohlsi bohlsi*	Mato Grosso do Sul, Brazil	100%	100%	0	KY304483
*groEL*	*Bartonella* sp.	*Hylaeamys* sp.; *Akodon* sp.	Ceará and Maranhão, Brazil	100%	97.46%	0	KX086735
*ftsZ*	*Bartonella* sp.	*Polygenis* (*P*.) *bohlsi bohlsi*	Mato Grosso do Sul, Brazil	100%	100%	0	KY304486
*rpoB*	*B. machadoae*	*Trichomys fosteri*	Mato Grosso do Sul, Brazil	95.54%	99%	0	CP087114
ITS	*B. machadoae*	*Trichomys fosteri*	Mato Grosso do Sul, Brazil	91.77%	32%	1 × 10^−52^	CP087114

**Table 3:** Phenotypic and genomic characteristics of *Bartonella harrusi* sp. nov.

Phenotypic Characterization	
Gram	Negative
Morphology	rods
Size	1–2.0 µm—0.3–0.6 µm
Growing conditions	Capnophilic, microaerophilic and aerobic
**Plasmid characterization**	
Total size	29.892 pb
%C + G	34.5%
**Genomic characterization**	
estimated completeness	99.35%
Total size	2.235.184 pb
%C + G	38.8%
Genes (total)	2013
CDSs (total)	1961
Genes (coding)	1737
CDSs (with proteins)	1737
Genes (RNA)	44
rRNAs	2, 2, 2 (5S, 16S, 23S)
rRNAs completes	2, 2, 2 (5S, 16S, 23S)
tRNAs	44
ncRNAs	4
Pseudo Genes (total)	224
CDSs (without proteins)	224
Pseudo Genes (ambiguous residues)	0 of 224
Pseudo Genes (frameshift)	144 of 224
Pseudo Genes (incomplete)	83 of 224
Pseudo Genes (stop internal)	68 of 224
Pseudo Genes (multiple problems)	63 of 224
Flagellum genes (*FlagA* and *FlagB*)	Absent
Adhesin genes (*BadA*)	Absent

**Table 4:** Percentage (%) of nucleotide identity between *Bartonella harrusi* sp. nov. and *B. machadoae* according to different molecular markers. The cut-off values proposed by [62] for description of new *Bartonella* species for each molecular marker are shown.

Target Gene Region	Identity	Query Cover	E-Value	Cut-Off Point [62]
16S rRNA	100%	100%	0.0	98.3%
*gltA*	94.53%	100%	0.0	93.6%
*groEL*	96.09%	100%	0.0	92.6%
*ftsZ*	92.84%	100%	0.0	94.4%
*rpoB*	95.54%	99%	0.0	92.8%
ITS	91.77%	32%	1 × 10^−52^	93.9%

## Data Availability

The data that support the findings of this study are openly available at the National Center for Biotechnology Information at https://www.ncbi.nlm.nih.gov/—accessed on 1 June 2022, reference number genome BioProject: PRJNA854809, genome accession numbers: CP101113-CP101114.

## Supplementary Materials

The following supporting information can be downloaded at: https://www.mdpi.com/article/10.3390/microorganisms10081609/s1. Figure S1. Gram-staining of *Bartonella harrusi* sp. nov. (strain 117A). Figure S2. Dendrogram created from the dRep analysis of all genomes deposited in GenBank using the 95% ANI value (average nucleotide identity) as a cut-off point by obtaining 50 different strains. Figure S3. Heat map generated in the search for genes related to metabolic profiles using the genomes of *Bartonella* species available in GenBank. Figure S4. Heat map generated in the search for genes related to ribosomal and polymerase phylogenomic markers for species of the genus *Bartonella.* Figure S5. Heat map generated in the search for families of active carbohydrate enzymes (CAZy) for species of the genus *Bartonella.* Table S1. Taxonomic reclassification of the *Bartonella* species based on the 95% cut-off point assessed by the Average Nucleotide Identity (ANI) value. *Bartonella* species with a new proposed taxonomic status are red marked. n/a: species that did not have ANI variation below the 97% cut-off.
