# Supplementary material for: First Report of Bartonella spp. in Marsupials from Brazil, with a Description of Bartonella harrusi sp. nov. and a New Proposal for the Taxonomic Reclassification of Species of the Genus Bartonella
Source: Microorganisms. 2022 Aug 9;10(8):1609. doi: 10.3390/microorganisms10081609 (PMC9414547; doi:10.3390/microorganisms10081609)
Supplement: Supplementary file 1 [file microorganisms-10-01609-s001.zip › Table S1.pdf]

Table S1. Taxonomic reclassification of the *Bartonella* species based on the 95% cut-off point assessed by the Average Nucleotide Identity (ANI) value. *Bartonella* species with a new proposed taxonomic status are red-marked. n/a: species that did not have ANI variation below the 97% cut-off.

Species on Genbank	Strain	Accession number	Lineage (based on cut off = 95%)	Subcluster (cut off = 97%)	New proposed classification
Bartonella bovis	CIP 106692	CACVBI010000001	1	subsp. 1	*Bartonella bovis* subsp. *1*
Bartonella bovis	91-4	CM001844	1	subsp. 1	*Bartonella bovis* subsp. *1*
Bartonella bovis	BbUM	MWVG01000001	1	subsp. 2	*Bartonella bovis* subsp. *2*
Bartonella bovis	m02	KB915624	1	subsp. 3	*Bartonella bovis* subsp. *3*
Bartonella sp.	WD12.1	MUBG01000001	2	n/a	*Bartonella* sp. nov. lineage 2
Bartonella schoenbuchensis	CCUG 50783	CADDYD010000001	3	subsp. 1	*Bartonella schoenbuchensis* subsp. *schoenbuchensis*
Bartonella schoenbuchensis	R1	CP019789	3	subsp. 1	*Bartonella schoenbuchensis* subsp. *schoenbuchensis*
Bartonella schoenbuchensis	m07a	KB915627	3	subsp. 1	*Bartonella schoenbuchensis* subsp. *schoembuchensis*
Bartonella chomelii	DSM 21431	NZ_JACJIR01000000 1	3	subsp. 1	*Bartonella schoenbuchensis* subsp. *schoembuchensis*
Bartonella capreoli	DSM 21569	CADDZX010000001	3	subsp. 2	*Bartonella schoenbuchensis* subsp. *capreoli*
Bartonella melophagi	K-2C	JH725081	3	subsp. 3	*Bartonella melophagi* subsp. *melophagi*
Bartonella sp.	WD16.2	CP019781	4	n/a	*Bartonella* sp. nov. lineage 4
Bartonella bacilliformis	USM-LMMB	LQWW01000001	5	subsp. 1	*Bartonella bacilliformis* subsp. *1*
Bartonella bacilliformis	USM-LMMB	LQXX01000001	5	subsp. 1	*Bartonella bacilliformis* subsp. *1*
Bartonella bacilliformis	ATCC 35685D-5	CP014012	5	subsp. 1	*Bartonella bacilliformis* subsp. *1*
Bartonella bacilliformis	INS	AMQK01000001	5	subsp. 1	*Bartonella bacilliformis* subsp. *1*
Bartonella bacilliformis	KC583	CP000524	5	subsp. 1	*Bartonella bacilliformis* subsp. *1*
Bartonella bacilliformis	Ver075	KL503822	5	subsp. 1	*Bartonella bacilliformis* subsp. *1*
Bartonella bacilliformis	CAR600-02	KL503826	5	subsp. 1	*Bartonella bacilliformis* subsp. *1*
Bartonella bacilliformis	VAB9028	KL503815	5	subsp. 1	*Bartonella bacilliformis* subsp. *1*
Bartonella bacilliformis	CUSCO5	KL503795	5	subsp. 1	*Bartonella bacilliformis* subsp. *1*
Bartonella bacilliformis	Cond044	KL503799	5	subsp. 1	*Bartonella bacilliformis* subsp. *1*
Bartonella bacilliformis	Peru38	KL503808	5	subsp. 1	*Bartonella bacilliformis* subsp. *1*
Bartonella bacilliformis	Hosp800-02	KL503779	5	subsp. 1	*Bartonella bacilliformis* subsp. *1*
Bartonella bacilliformis	Heidi	KK097685	5	subsp. 1	*Bartonella bacilliformis* subsp. *1*
Bartonella bacilliformis	San Pedro600-02	KK097680	5	subsp. 1	*Bartonella bacilliformis* subsp. *1*
Bartonella bacilliformis	Peru-18	KK097689	5	subsp. 1	*Bartonella bacilliformis* subsp. *1*
Bartonella bacilliformis	Ver097	KL503802	5	subsp. 2	*Bartonella bacilliformis* subsp. *2*
Bartonella australis	Aust/NH1	CP003123	6	n/a	*Bartonella australis*
Bartonella elizabethae	NCTC12898	LR134527	7	subsp. 1	*Bartonella elizabethae* subsp.. *1*
Bartonella elizabethae	BeUM	LFMF01000001	7	subsp. 1	*Bartonella elizabethae* subsp.. *1*
Bartonella elizabethae	F9251 = ATCC	JADB01000001	7	subsp. 1	*Bartonella elizabethae* subsp.. *1*
Bartonella elizabethae	F9251	JH725033	7	subsp. 1	*Bartonella elizabethae* subsp.. *1*
Bartonella elizabethae	Re6043vi	JH725139	7	subsp. 1	*Bartonella elizabethae* subsp.. *1*
Bartonella elizabethae	F9251 = ATCC	CADEAC010000001	7	subsp. 1	*Bartonella elizabethae* subsp.. *1*
Bartonella sp.	.008	LT883127	7	subsp. 2	*Bartonella elizabethae* subsp. *2*
Bartonella krasnovii	B60_6	CP093035	8	subsp. 1	*Bartonella krasnovii* subsp. *1*
Bartonella krasnovii	B84_4	CP093038	8	subsp. 1	*Bartonella krasnovii* subsp. *1*
Bartonella krasnovii	84B_2	CP093046	8	subsp. 1	*Bartonella krasnovii* subsp. *1*
Bartonella krasnovii	75A_1b	CP093040	8	subsp. 1	*Bartonella krasnovii* subsp. *1*
Bartonella krasnovii	90B_7	CP093044	8	subsp. 1	*Bartonella krasnovii* subsp. *1*
Bartonella krasnovii	51A_7	CP093043	8	subsp. 1	*Bartonella krasnovii* subsp. *1*
Bartonella krasnovii	B9_5	CP093039	8	subsp. 1	*Bartonella krasnovii* subsp. *1*
Bartonella krasnovii	75A_4b	CP093042	8	subsp. 1	*Bartonella krasnovii* subsp. *1*
Bartonella krasnovii	87B_3	CP093041	8	subsp. 1	*Bartonella krasnovii* subsp. *1*
Bartonella krasnovii	B1_2	CP093037	8	subsp. 1	*Bartonella krasnovii* subsp. *1*
Bartonella krasnovii	B71	CP093034	8	subsp. 1	*Bartonella krasnovii* subsp. *1*
Bartonella krasnovii	B35_1_2	CP093033	8	subsp. 1	*Bartonella krasnovii* subsp. *1*
Bartonella krasnovii	B51_4	CP093036	8	subsp. 1	*Bartonella krasnovii* subsp. *1*
Bartonella krasnovii	OE 1-1	CP031844	8	subsp. 2	*Bartonella krasnovii* subsp. *2*
Bartonella krasnovii	36C_7b	CP093045	8	subsp. 3	*Bartonella krasnovii* subsp. *3*
Candidatus Bartonella saheliensis	.077	LR607310	9	n/a	*Bartonella saheliensis* sp. nov.
Bartonella grahamii	ATCC 700132	JACX01000001	10	subsp. 1	*Bartonella grahamii* subsp. *1*

Bartonella grahamii	NCTC12860	UFTD01000005	10	subsp. 1	*Bartonella grahamii* subsp. *1*
Bartonella grahamii	as4aup	CP001562	10	subsp. 2	*Bartonella grahamii* subsp. *2*
Bartonella grahamii	A7JPB	CACVBG010000001	10	subsp. 3	*Bartonella grahamii* subsp. *3*
Bartonella grahamii	A1JPB	CACVBJ010000001	10	subsp. 4	*Bartonella grahamii* subsp. *4*
Bartonella tribocorum	L103	CADDYI010000001	11	n/a	*Bartonella* sp. nov. lineage *11*
Bartonella tribocorum	L103	NJGE01000001	11	n/a	*Bartonella* sp. nov. lineage *11*
Bartonella tribocorum	CIP 105476	AM260525	12	n/a	*Bartonella tribocorum*
Bartonella tribocorum	BM1374166	CADDYK010000001	12	n/a	*Bartonella tribocorum*
Bartonella kosoyi	Tel Aviv	CP031843	13	n/a	*Bartonella kosoyi*
Bartonella tribocorum	C635	NJPP01000001	14	n/a	*Bartonella* sp. nov. lineage *14*
Bartonella tribocorum	C635	CADDYJ010000001	14	n/a	*Bartonella* sp. nov. lineage *14*
Bartonella queenslandensis	AUST/NH15	HE997969	15	subsp. 1	*Bartonella queenslandensis* subsp. *1*
Bartonella queenslandensis	BqUM	NZ_NAAI01000001	15	subsp. 2	*Bartonella queenslandensis* subsp. *2*
Bartonella massiliensis	OS09	CABFVS010000001	16	n/a	*Bartonella massiliensis*
Bartonella rattimassiliensis	15908	CALY02000093	17	n/a	*Bartonella rattimassiliensis*
Bartonella rattimassiliensis	15908	JH725064	17	n/a	*Bartonella rattimassiliensis*
Bartonella fuyuanensis	DSM 100694	JACIFE010000001	18	n/a	*Bartonella fuyuanensis*
Bartonella koehlerae	C-29	KL407334	19	n/a	*Bartonella koehlerae*
Bartonella koehlerae	CCUG 50773	CADEAH010000001	19	n/a	*Bartonella koehlerae*
Bartonella henselae	BM1374164	CACVBL010000001	20	n/a	*Bartonella henselae*
Bartonella henselae	BM1374163	CACVBD010000001	20	n/a	*Bartonella henselae*
Bartonella henselae	BM1374165	CACVBK010000001	20	n/a	*Bartonella henselae*
Bartonella henselae	ATCC49882T var-1	CP072903	20	n/a	*Bartonella henselae*
Bartonella henselae	ATCC49882T	CP072902	20	n/a	*Bartonella henselae*
Bartonella henselae	Marseille	CP072904	20	n/a	*Bartonella henselae*
Bartonella henselae	88-64	CP072899	20	n/a	*Bartonella henselae*
Bartonella henselae	FR96/BK38	CP072898	20	n/a	*Bartonella henselae*
Bartonella henselae	G-5436	CP072900	20	n/a	*Bartonella henselae*
Bartonella henselae	FR96/BK3	CP072897	20	n/a	*Bartonella henselae*
Bartonella henselae	FDAARGOS_1462	CP082885	20	n/a	*Bartonella henselae*
Bartonella henselae	Berlin-I	CP072901	20	n/a	*Bartonella henselae*
Bartonella henselae	623-125	BLJS01000001	20	n/a	*Bartonella henselae*
Bartonella henselae	804-29	BLJT01000001	20	n/a	*Bartonella henselae*
Bartonella henselae	Houston-I	CP020742	20	n/a	*Bartonella henselae*
Bartonella henselae	A242	LOAF01000001	20	n/a	*Bartonella henselae*
Bartonella henselae	A121	LOAC01000001	20	n/a	*Bartonella henselae*
Bartonella henselae	F1	LOAI01000001	20	n/a	*Bartonella henselae*
Bartonella henselae	A244	LOAG01000001	20	n/a	*Bartonella henselae*
Bartonella henselae	FR96/BK3	CP072897	20	n/a	*Bartonella henselae*
Bartonella henselae	A112	LOAB01000001	20	n/a	*Bartonella henselae*
Bartonella henselae	A235	LOAE01000001	20	n/a	*Bartonella henselae*
Bartonella henselae	A71	LOAA01000001	20	n/a	*Bartonella henselae*
Bartonella henselae	A233	LOAD01000001	20	n/a	*Bartonella henselae*
Bartonella henselae	A20	LNZX01000001	20	n/a	*Bartonella henselae*
Bartonella henselae	Houston-1	LRIJ02000001	20	n/a	*Bartonella henselae*
Bartonella henselae	A76	LNZZ01000001	20	n/a	*Bartonella henselae*
Bartonella henselae	A74	LNZY01000001	20	n/a	*Bartonella henselae*
Bartonella henselae	MVT02	LN879429	20	n/a	*Bartonella henselae*
Bartonella henselae	BM1374163	HG965802	20	n/a	*Bartonella henselae*
Bartonella henselae	BM1374165	HG969191	20	n/a	*Bartonella henselae*
Bartonella henselae	Houston-1	BX897699	20	n/a	*Bartonella henselae*
Bartonella henselae	Zeus	KL411698	20	n/a	*Bartonella henselae*
Bartonella henselae	JK 53	KL411717	20	n/a	*Bartonella henselae*
Bartonella henselae	JK 41	KI911811	20	n/a	*Bartonella henselae*
Bartonella henselae	JK 42	KI911798	20	n/a	*Bartonella henselae*
Bartonella henselae	JK 50	KI911793	20	n/a	*Bartonella henselae*
Bartonella henselae	JK 51	KI911786	20	n/a	*Bartonella henselae*
Bartonella senegalensis	OS02	NZ_HE997540	21	n/a	*Bartonella senegalensis*
Bartonella callosciuri	DSM 28538	JACHIM010000001	22	n/a	*Bartonella callosciuri*
Bartonella phoceensis	CIP107707	CADEAD010000001	23	n/a	*Bartonella phoceensis*
Bartonella quintana	CCUG 45777	CADDYB010000001	24	n/a	*Bartonella quintana*
Bartonella quintana	NCTC12899	LS483373	24	n/a	*Bartonella quintana*
Bartonella quintana	G1712	CP091505	24	n/a	*Bartonella quintana*
Bartonella quintana	G1713	CP091504	24	n/a	*Bartonella quintana*
Bartonella quintana	KorN	CP041670	24	n/a	*Bartonella quintana*
Bartonella quintana	MF1-1	AP019773	24	n/a	*Bartonella quintana*
Bartonella quintana	RM-11	CP003784	24	n/a	*Bartonella quintana*
Bartonella quintana	Toulouse	BX897700	24	n/a	*Bartonella quintana*
Bartonella quintana	JK 68	KL446932	24	n/a	*Bartonella quintana*

Bartonella quintana	JK 39	KL446941	24	n/a	*Bartonella quintana*
Bartonella quintana	JK 56	KL446928	24	n/a	*Bartonella quintana*
Bartonella quintana	JK 67	KL446937	24	n/a	*Bartonella quintana*
Bartonella quintana	JK 63	KL411737	24	n/a	*Bartonella quintana*
Bartonella quintana	JK 31	KL411732	24	n/a	*Bartonella quintana*
Bartonella quintana	JK 19	KL411729	24	n/a	*Bartonella quintana*
Bartonella quintana	BQ2-D70	KI911820	24	n/a	*Bartonella quintana*
Bartonella quintana	JK 7	KI911831	24	n/a	*Bartonella quintana*
Bartonella quintana	JK 73	KI911825	24	n/a	*Bartonella quintana*
Bartonella quintana	JK 73rel	KI911823	24	n/a	*Bartonella quintana*
Bartonella quintana	JK 12	KI911828	24	n/a	*Bartonella quintana*
Bartonella washoensis	NCTC13399	UAQI01000043	25	n/a	*Bartonella washoeensis*
Bartonella washoensis	085-0475	JH725101	25	n/a	*Bartonella washoeensis*
Bartonella washoensis	Sb944nv	JH725022	25	n/a	*Bartonella washoeensis*
Bartonella machadoae	56A	CP087114	26	n/a	*Bartonella machadoae*
Bartonella harrusi sp. nov.	117A		27	n/a	*Bartonella harrusi* sp. nov.
Bartonella vinsonii subsp. *arupensis*	ATCC 700727	CADEAE010000001	28	n/a	*Bartonella arupensis*
Bartonella vinsonii subsp. *arupensis*	Pm136co	JH725043	28	n/a	*Bartonella arupensis*
Bartonella vinsonii subsp. *arupensis*	OK-94-513	JH725037	28	n/a	*Bartonella arupensis*
Bartonella vinsonii	NCTC12905	LR134529	29	n/a	*Bartonella vinsonii*
Bartonella vinsonii subsp. *vinsonii*	CIP 103738	CADEAJ010000001	29	n/a	*Bartonella vinsonii*
Bartonella vinsonii subsp. *berkhoffii*	Winnie	CP003124	30	n/a	*Bartonella berkhoffii*
Bartonella vinsonii subsp. *berkhoffii*	ATCC	JACY01000001	30	n/a	*Bartonella berkhoffii*
Bartonella vinsonii subsp. *berkhoffii*	Tweed	KB915630	30	n/a	*Bartonella berkhoffii*
Bartonella vinsonii subsp. *berkhoffii*	CIP 104960	CADEAK010000001	30	n/a	*Bartonella berkhoffii*
Bartonella doshiae	CCUG 50770	CACVBH010000001	31	n/a	*Bartonella doshiae*
Bartonella doshiae	A14JPB	CACVBC010000001	31	n/a	*Bartonella doshiae*
Bartonella doshiae	BM1374167	CACVBF010000001	31	n/a	*Bartonella doshiae*
Bartonella doshiae	SRS24	CACVBE010000001	31	n/a	*Bartonella doshiae*
Bartonella doshiae	NCTC12862	UFTF01000002	31	n/a	*Bartonella doshiae*
Bartonella doshiae	BM1374167	CCBL010000001	31	n/a	*Bartonella doshiae*
Bartonella doshiae	DSM 102055	JACHEI010000001	31	n/a	*Bartonella doshiae*
Bartonella doshiae	NCTC 12862 = ATCC	JAGY01000001	31	n/a	*Bartonella doshiae*
Bartonella doshiae	NCTC 12862	JH725094	31	n/a	*Bartonella doshiae*
Bartonella birtlesii	IBS 325	CM001557	32	n/a	*Bartonella birtlesii*
Bartonella birtlesii	E4	KE007216	32	n/a	*Bartonella birtlesii*
Bartonella birtlesii	E11	KE007210	32	n/a	*Bartonella birtlesii*
Bartonella birtlesii	E7	KE007202	32	n/a	*Bartonella birtlesii*
Bartonella birtlesii	LL-WM9	JH725076	32	n/a	*Bartonella birtlesii*
Bartonella florencae	R4	HE997451	33	n/a	*Bartonella florencae*
Bartonella alsatica	IBS 382T	CACVBB010000001	34	n/a	*Bartonella alsatica*
Bartonella alsatica	CIP 105477	CP058235	34	n/a	*Bartonella alsatica*
Bartonella alsatica	IBS 382	JH725020	34	n/a	*Bartonella alsatica*
Bartonella sp.	B191	LR736368	35	subsp. 1	*Bartonella* sp. nov. lineage *35* subsp. *1*
Bartonella sp.	DB5-6	JH725114	35	subsp. 2	*Bartonella* sp. nov. lineage *35* subsp. *2*
Bartonella taylorii	A12JPB	CADDYH010000001	36	subsp. 1	*Bartonella taylorii* subsp. *1*
Bartonella taylorii	SRS19	CADDYE010000001	36	subsp. 1	*Bartonella taylorii* subsp. *1*
Bartonella taylorii	8TBB	JH725050	36	subsp. 1	*Bartonella taylorii* subsp. *1*
Bartonella taylorii	SRS10	CADDYG010000001	36	subsp. 2	*Bartonella taylorii* subsp. *2*
Bartonella taylorii	SRS29	CADEAG010000001	36	subsp. 3	*Bartonella taylorii* subsp. *3*
Candidatus Bartonella raoultii	.094	JAIFRO010000010	37	n/a	*Bartonella raoultii* sp. nov.
Bartonella rattaustraliani	AUST/NH4	CALW02000108	38	n/a	*Bartonella rattaustraliani*
Bartonella tamiae	Th307	JH725021	39	n/a	*Bartonella tamiae*
Bartonella tamiae	Th239	JH725147	39	n/a	*Bartonella tamiae*
Bartonella sp.	HY038	CP059725	40	n/a	*Bartonella* sp. lineage *40*
Bartonella sp.	B10834G3	JACFOM010000009	41	n/a	*Bartonella* sp. lineage *41*
Bartonella sp.	B10834H15	JACFOL010000031	41	n/a	*Bartonella* sp. lineage *41*
Bartonella sp.	W8125	JACFRR010000008	41	n/a	*Bartonella* sp. lineage *41*
Bartonella apis	BBC0122	CP015625	41	n/a	*Bartonella* sp. lineage *41*
Bartonella sp.	B10834G6	JACFOX010000008	42	subsp. 1	*Bartonella* sp. lineage *42* subsp. *1*
Bartonella sp.	W8099	JACFSN010000007	42	subsp. 1	*Bartonella* sp. lineage *42* subsp. *1*
Bartonella apis	PEB0122	LXYU01000009	42	subsp. 2	*Bartonella* sp. lineage *42* subsp. *2*

Bartonella apis	PEB0150	LXYS01000009	42	subsp. 2	*Bartonella* sp. lineage *42* subsp. *2*
Bartonella apis	PEB0149	LXYT01000007	42	subsp. 2	*Bartonella* sp. lineage *42* subsp. *2*
Bartonella sp.	W8098	JACFOW010000007	42	subsp. 3	*Bartonella* sp. lineage *42* subsp. *3*
Bartonella sp.	W8152	JACFSP010000008	42	subsp. 3	*Bartonella* sp. lineage *42* subsp. *3*
Bartonella sp.	W8151	JACFSO010000007	42	subsp. 3	*Bartonella* sp. lineage *42* subsp. *3*
Bartonella sp.	W8167	JACFSQ010000017	42	subsp. 3	*Bartonella* sp. lineage *42* subsp. *3*
Bartonella apis	BBC0244	CP015821	43	n/a	*Bartonella apis*
Bartonella sp.	BBC0178	CP015820	43	n/a	*Bartonella apis*
Bartonella apis	M0190	JACFOS010000003	43	n/a	*Bartonella apis*
Bartonella sp.	M0187	JACFOR010000010	43	n/a	*Bartonella apis*
Bartonella sp.	W8122	JACFOO010000008	43	n/a	*Bartonella apis*
Bartonella sp.	W8097	JACFON010000008	43	n/a	*Bartonella apis*
Bartonella sp.	P0291	JACFOQ010000001	43	n/a	*Bartonella apis*
Bartonella sp.	M0176	JACFOP010000004	43	n/a	*Bartonella apis*
Bartonella sp.	M0193	JACFOV010000087	43	n/a	*Bartonella apis*
Bartonella sp.	M0192	JACFOU010000003	43	n/a	*Bartonella apis*
Bartonella sp.	M0191	JACFOT010000007	43	n/a	*Bartonella apis*
Bartonella sp.	M0280	JACFSJ010000008	43	n/a	*Bartonella apis*
Bartonella sp.	M0177	JACFOY010000001	44	n/a	*Bartonella* sp. nov. lineage *44*
Bartonella sp.	M0283	JACFSK010000001	45	n/a	*Bartonella* sp. nov. lineage *45*
Bartonella sp.	JB15	CP019787	46		*Bartonella* sp. nov. lineage *46*
Bartonella sp.	JB63	CP019788	46		*Bartonella* sp. nov. lineage *46*
Bartonella clarridgeiae	73	FN645454	47	n/a	*Bartonella clarridgeiae*
Bartonella clarridgeiae	ATCC 51734	JADC01000009	47	n/a	*Bartonella clarridgeiae*
Bartonella sp.	114	CP019784	48	subsp. 1	*Bartonella rochalimae* subsp. *1*
Bartonella sp.	11B	CP019783	48	subsp. 1	*Bartonella rochalimae* subsp. *1*
Bartonella sp.	Raccoon60	CP019786	48	subsp. 1	*Bartonella rochalimae* subsp. *1*
Bartonella sp.	CDC_skunk	CP019782	48	subsp. 1	*Bartonella rochalimae* subsp. *1*
Bartonella sp.	A1379B	CP019780	48	subsp. 1	*Bartonella rochalimae* subsp. *1*
Bartonella rochalimae	ATCC BAA-1498	KL407337	48	subsp. 1	*Bartonella rochalimae* subsp. *1*
Bartonella sp.	1-1C	CP019489	48	subsp. 2	*Bartonella rochalimae* subsp. *2*
Bartonella sp.	Ga0114335_11	MUYE01000001	49	n/a	*Bartonella* sp. nov. lineage *49*
Bartonella ancashensis	20.00	CP010401	50	n/a	*Bartonella ancashensis*
