# Supplementary figures and images for: First Report of Bartonella spp. in Marsupials from Brazil, with a Description of Bartonella harrusi sp. nov. and a New Proposal for the Taxonomic Reclassification of Species of the Genus Bartonella
Source: Microorganisms. 2022 Aug 9;10(8):1609. doi: 10.3390/microorganisms10081609 (PMC9414547; doi:10.3390/microorganisms10081609)

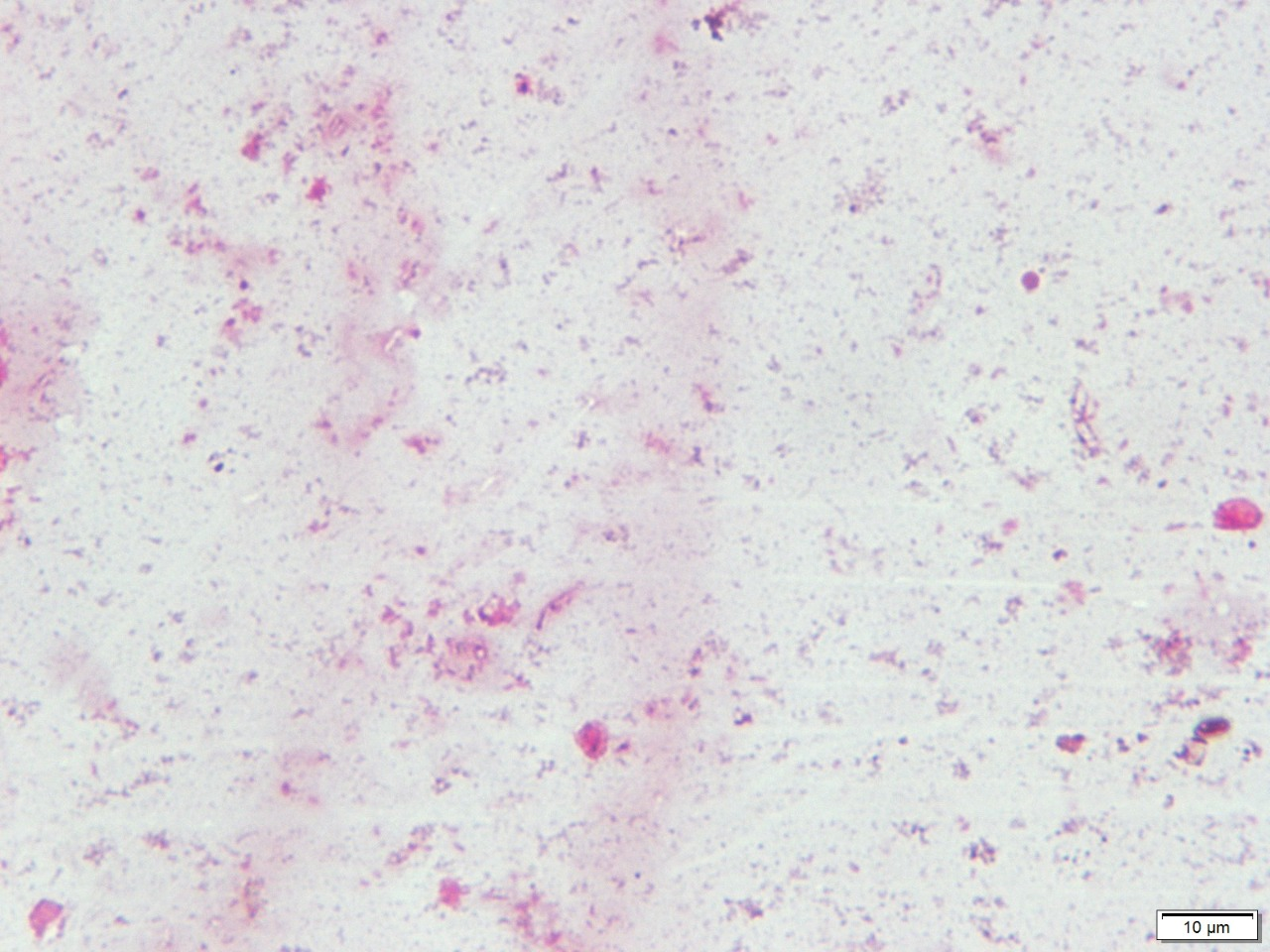

Supplement: Supplementary file 1 [file microorganisms-10-01609-s001.zip › Figure S1.tif]

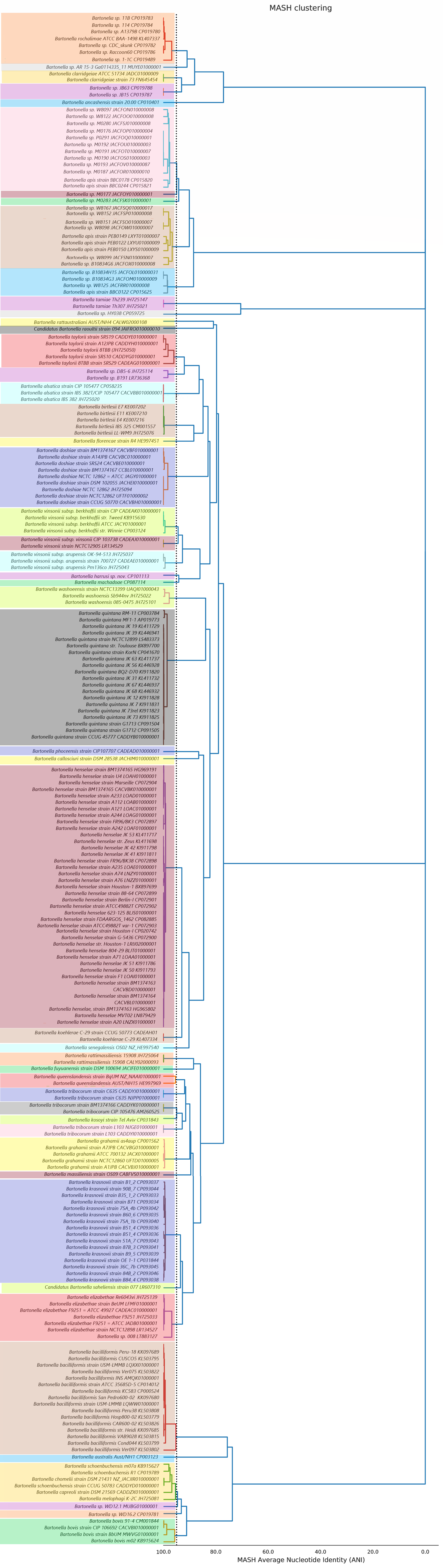

Supplement: Supplementary file 1 [file microorganisms-10-01609-s001.zip › Figure S2.tiff]
